# The Impact of Serialisation on Operational Efficiency and Productivity in Irish Pharmaceutical Sites

**DOI:** 10.1007/s43441-024-00662-1

**Published:** 2024-05-29

**Authors:** Daniel O’ Mahony, Alan Lynch, Olivia McDermott

**Affiliations:** 1 College of Science and Engineering, University of Galway, Galway, Ireland; 2 Faculty of Science, Griffith College, Dublin, Ireland; 3 College of Science and Engineering, University of Galway, Galway, Ireland

**Keywords:** Pharmaceutical COGS, Overall Equipment Effectiveness, Serialisation

## Abstract

Technology enabling drug serialisation technology was introduced by regulators to enhance security in pharmaceutical supply chain and protect drugs from infiltration by falsified and substandard medicines. The introduction of systems for serialisation required huge financial outlays manufacturers of pharmaceuticals. This study investigated the impact of serialisation on the operational efficiency and productivity in Irish pharmaceutical sites. A qualitative study was conducted with 11 manufacturing sites in Ireland. The participating companies operated a total of 114 pack-lines, representing approximately 65% of the automated packing lines in the country. The study found that serialisation had a negative effect on packaging production line OEE and line availability and on the individuals cost per unit of packaged pharmaceuticals. The research results estimated that the capital costs of serialisation were four times greater than those estimated by the regulators. There was a 4.1 cents average cost per pack for serialisation with high volume sites reporting an annual cost of serialisation of up to €4.5 m per annum and a 2.7% increase in the average cost of goods sold. A pattern whereby where many pharmaceutical manufacturers are transitioning from smaller batch production and moving toward larger batch production sizes in order to increases efficiencies was identified. The research also proposed the use of a serialisation depreciation factor as a method to determine the impact of serialisation on the cost of goods sold. This is the first study of its kind into the cost of serialisation from a manufacturer’s viewpoint and studying the effects of serialisation on productivity, line availability and operational efficiency.

## Introduction

In recent years the World Health Organization (WHO) has communicated a security issue in relation to the public from the use and availability of falsified and substandard medicines, and these medicines have infiltrated the legitimate supply chain [1]. Due to incidents with patient injury and death because of falsified medicines rising dramatically, globally based authorities drew up legislation in an effort to protect the public [2, 3]. The introduction of anti-counterfeiting regulations such as serialisation necessitates a large financial outlay by pharmaceutical manufacturers in investing and purchasing in new technology [4]. The regulations mandated the use of serialisation to deploy a unique serial identifier on each unit of packaging of a produced medicine [5]. Every carton, bottle or vial of medicine produced for the American and EU markets have to contain a serialised code that is unique to that pack with a serial code, expiry date and batch number are contained in a 2D matrix code mandated by the regulators [6]

While to print and check a simple code is easy for one unit, to print without error for example 1200 codes per hour of a 24-h manufacturing shift requires a great deal of skill and resources. Serialisation at a unit level involves the creation of a lot of data that must be correctly stored, has the ability to be retrieved when required, and shared between several systems. Any single pharmaceutical manufacturer can generate up to 700 GB of data from its serialisation processes per annum [7]. Any issues with creating this data, retrieving and communicating the data could rests in manufacturing line delays or even stoppages, may lead to a product recall and affect medicine supply to patients [8]. The European Federation of Pharmaceutical Industries and Associations (EFPIA) report referenced a total annual cost to the pharmaceutical industry of €125 m for serialisation.

There were many benefits to the Pharma industry in terms of serialisation by improving costs of inventory management, in reducing wastage and disposal costs caused by expired products; improved inventory accuracy via traceability and recognition of the expiration date of the product and enhancing recall capacity and monitoring of drugs [9].

Irish based pharmaceutical companies must meet the regulatory demands of all the markets supplied from Ireland as the majority of Ireland’s 2019 exports (€39bn) going to the U.S. market are made up of pharmaceutical products [10]. All ten of the global top ten pharmaceutical companies have manufacturing operations in Ireland [11]. The value of pharmaceutical exports from Ireland in 2018 was €73billion [12]. The Irish competent authority the Health Products Regulatory Authority (HPRA) oversees pharmaceutical manufacturing activities in Ireland. Irish based manufacturers must meet Good Manufacturing Practice (GMP) and quality management standards of all the markets served including the U.S. Food and Drug Administration (FDA) and European Medicines Agency (EMA).

The purpose of the study is to investigate the effect of serialisation on Irish pharmaceutical manufacturing lines in terms of efficiency and productivity. Serialisation inherently requires addition of new process steps into existing operations which can affect operations efficiency metrics [8, 13]. Serialisation required a substantial investment by the pharmaceutical industry in terms of capital expenditure and may result in the need for specific expertise and additional personnel to aid with the deployment and maintenance of serialisation systems. The research questions (RQ’s) are thus:

- Identify what were the effects of serialisation on operations lines and their productivity in the process of implementing serialisation and after the implementation of serialisation processes.
- Quantify the costs of serialisation on pharmaceutical manufacturing lines.

Section 2 outlines the literature review, Sect. 3 the methodology while the results of the study are outlined in Sect. 4. Section 5 and 6 summarises the discussion and conclusion.

## Literature Review

### Background to Serialisation

Analysis has been carried out to ascertain the effects of counterfeit medicines in the European Union. In a 2020 report the European Union Intellectual Property Organisation (EUIPO) and the Organization for Economic Cooperation and Development (OECD) found that the costs of counterfeit drugs were nearly 5 billion euro globally. Their report further stated that nearly 40% of counterfeit drugs seized were infringing basic trademarks and patents [14, 15]. It was also established that there were unlicensed medicines which were contributing to job losses of nearly 90,000 personnel directly within and around the supporting pharma industry with a total cost of over ten billion euro in lost revenue from counterfeit medication [15]. There is further a human cost to having fake medicines circulated. According to the EUIPO/OECD report, up to 170,000 children have succumbed to pneumonia from taking counterfeit and poor-quality antibiotics falsified. Thus there was a huge impetus to regulate to provide more vigilance around medicine availability and movements and clamp down on any illegal activities [1].

Pharmaceutical supply chains are long and can be more complicated than other types of supply chains [3]. Systems that track pharmaceuticals must contain several data types on the drug label: country of origin ID, Manufacturers ID, and National Drug Code, which is very suitable for serialisation. The ISO 16022 was published in 2006 as the international standard for the use of 2D data matrix codes [13]. A further standard on how to grade the print quality of 2D Data matrix codes was also published [14] aiding the application of 2D data matrix codes by the pharmaceutical industry [].

#### The Expected Costs of Serialisation

The EFPIA reported an annualised cost of €125 m to the pharma industry in relation to serialisation but did not specify where exactly in the supply chain these costs would be spent [17]. They did state that there would be 1.6 cent added per pack of medicine with annual costs to the manufacturer of €8 m based on an average $7bn in sales and a production of half a million packs a year [17].

Out of the 100 responses made to the European Commission during the consultation process on counterfeit medicines, none of them took into account the potential effects on manufacturing efficiency and the subsequent impact on the supply of medicines due to serialization. The analysis approximates that the one-time expenses for implementing serialisation technology would amount to €150,000 per packaging line. Upgrading the 12,000 non-prescription pharmaceuticals pack lines will require an industry expenditure of €1.8bn. Furthermore, an additional €4bn investment was necessary to establish the essential IT systems for effectively managing the transmission of serialized data. According to the final report, the projected cost for printing and packing serialized codes is 2 cents per box throughout the initial five-year period. After 5 years, the price per pack decreased to half a cent, perhaps as a result of equipment depreciation. Considering the yearly trade of 14.85 billion packs of prescription medications in the EU, the cost of printing and verifying serialisation codes on European pack lines amounts to €297 million per year, which is equivalent to 2 cents each pack [19].

In the United States, the FDA did not conduct a direct impact assessment, but there were various indirect reports that evaluated the potential effects of serialization processes on the industry. According to the Pew Healthcare research, the average cost to implement serialization on a pack line is estimated to be $1.4 million, as reported by pharmaceutical companies and vendors []. This cost encompasses not only the expenses associated with equipment and software, but also the expenses related to project implementation and company costs. Furthermore, it exceeded the estimations provided by the European Commission. The analysis indicates an increase in labour expenses up to $291,000 per year, per pack line. However, it does not mention any effect on operational efficiency as stated in the report [20].

The U.S. Center for Disease Control (CDC) published an effect assessment analysis on the printing of 2D data matrix codes on the vaccines supply chain. The report highlighted the increased complexity of printing 2D matrix codes in comparison to typical linear barcodes [21]. The regulations require producers to attain a minimum International Standards Organisation (ISO) grade C for printed labels, and each label must undergo verification to verify its legibility. Barcode scanners are insufficiently fast to read all the labels on a high-speed pack line, necessitating the use of industrial grade cameras [8]. In addition to the intricacy of the 2D codes, the FDA mandated that manufacturers must continue to include linear barcodes on packaging, which therefore raises the likelihood of printing mistakes [22]. Unfortunately, the impact study from the Center for Drug Control (CDC) did not mention the potential effect of serialization on operational efficiency.

### Efficiency in Manufacturing

The OEE approach (Overall Equipment Effectiveness) is commonly used in manufacturing environments to assess operational efficiency. Nakijima [23] discovered six parameters that have the most significant influence on Overall Equipment Effectiveness (OEE). These elements are: Losses resulting from equipment failure or breakdown, 2. The setup/adjustment time is 3 units. Idle and minor stoppage losses, 4. Line speed decreased to 5. Yield is currently decreased till the machines stabilize and reach a stable state. High quality. Serialisation has the capacity to impact the six parameters that influence Overall Equipment Effectiveness (OEE), as well as the three components that constitute the OEE calculation. The need to print intricate 2D matrix codes, affix tamper-evident seals, and verify the legibility of the print in accordance with ISO standards may impede the performance of the packaging line speed. The availability of the production line may be impacted by the duration required for operators to configure serialization data, remove unnecessary serialized codes, and the interruptions caused by substandard print quality [24]. 2D Matrix Codes, also known as data matrix codes (DMC), have the advantage of being legible from any direction. They also contain a built-in error correction feature, which enables them to be properly read even if the print quality is degraded by up to 30%. 2D data matrix codes contribute to maximizing Overall Equipment Effectiveness (OEE) from a quality perspective.

According to Rotunno et al. [25], the implementation of serialisation could have an influence on the overall equipment effectiveness (OEE) of the manufacturing line due to the necessary operational adjustments. The authors asserted that the continuous exchange of data between various components, synchronization, and the need to wait for valid data signals may lead to a decrease in overall line speed, resulting in a loss of performance efficiency. However, the Rotunno study did not measure the specific impact on pack line efficiency.

The Serialization Playbook, published by Healthcare Packaging, estimated that the deployment would result in a negative impact on Overall Equipment Effectiveness (OEE) ranging from 8 to 10%. The research projected that the Overall Equipment Effectiveness (OEE) would improve but still remain 4% below the level before the implementation of serialisation, which was at 29. The range of OEE loss, as determined by Ozkaya et al. [27] in a study published in Pharmaceutical Commerce, was found to be between 5 and 10% during the period following ramp-up and stabilization. Nevertheless, several organizations experienced losses of up to 30% during the initial phase of increasing activity following the implementation [27]. The paper also emphasized that operators would require training and expertise in order to optimize efficiency after the implementation of serialisation. The International Society of Pharmaceutical Engineers (ISPE) conducted a study on the losses in Overall Equipment Effectiveness (OEE) caused by the deployment of serialisation. According to the ISPE paper, losses ranging from 10 to 25% were reported by industry sources over the two months following the deployment of serialisation. After around six months, the lines may experience a decline of 1% to 5% from their original OEE position [13].

Whenever a serialisation system is modified to comply with these regulatory demands, the packing line must be halted, hence impacting the productivity of the manufacturing facility [8].

The phenomenon of Operational Excellence in the pharmaceutical industry.

The pharmaceutical business was slow to adopt operational excellence practices. The pharmaceutical sector demonstrated a clear indication of this through its substantial amounts of raw materials and completed inventory, in comparison to other industries []. The Hatch–Waxman Act, enacted in 1984, established the framework for generic medication makers to enter the market and compete with branded drug firms after the expiration of a medicine’s patent protection. The decrease in profit margins caused by generic manufacturers created a pressing need for pharmaceutical corporations to implement improvements. In order to participate in markets that do not have patent protection, producers were required to implement Lean manufacturing techniques [30]. The implementation of manufacturing licenses by regulators was frequently mentioned as a deterrent for pharmaceutical enterprises to enhance their operations [31]. The processes were perceived as stagnant and resistant to enhancement [32, 33]. Since the 2000s, the pharmaceutical industry has used operational excellence methodologies, resulting in significant enhancements in Overall Equipment Effectiveness (OEE) and other important performance metrics [29]. In 2007, the average Overall Equipment Efficiency (OEE) in a top-performing food processing operation was 24% higher than the average OEE in a top-performing pharmaceutical company [34, 35]. Pharmaceutical companies face challenges in achieving OEE (Overall Equipment Efficiency) compared to other industry sectors, mostly because of the time it takes to switch between batches. Pharmaceutical regulations require enterprises to thoroughly clean packing lines between batches in order to prevent product mix-ups and contamination [36]. Prior to manufacturing, it is necessary to establish checks for batch number, expiry date, and serialisation information on the pack lines. At each stage of the process, it is crucial to meticulously verify and cross-reference against standard operating procedures (SOPs) as required by Good Manufacturing Practices (GMP) [33]. The top-performing pharmaceutical companies achieved a four-fold decrease in changeover times compared to the lowest-performing sites [38]. The implementation of just-in-time manufacturing, along with the production of build-to-order batches, results in a higher occurrence of changeovers. This increase in changeovers has a detrimental effect on line availability and overall equipment effectiveness (OEE) [39, 40]. The procedure of setting up the serialisation can directly impact the duration of these changeover times. Any postponement in establishing serialization data for a batch during batch changeover has an effect on availability. As the number of batches processed on a pack line increases, the likelihood of errors occurring in the serialisation label print and check systems also increases, which can negatively impact the quality of the product.

The adverse consequences on Overall Equipment Effectiveness (OEE) must be counterbalanced with the favorable outcomes of implementing serialization. The age of the pack line can potentially affect this balance. The typical lifespan of pack line equipment is typically 20 to 25 years [41]. With the introduction of serialisation, certain manufacturers may have chosen to replace their older packaging lines with more modern equipment. This capital expenditure investment in new equipment may have resulted in improved line speeds and quicker changeover times. The installation of better cameras and printers during a serialisation process can enhance line performance, even without replacing older pack lines. According to one vendor, a factory was able to save $100,000 USD per year by substituting hand inspectors with an automated vision system during the installation of a serialisation process []. According to another vendor, a client has begun closely monitoring OEE (Overall Equipment Efficiency) after implementing serialisation. They are working closely with operators to reduce waste and have successfully increased OEE by 20% [42].

The pharmaceutical pack line represents the final phase of customization in the manufacturing process. A substance is not considered a drug until it is appropriately labeled and serialized for a particular market. Therefore, any influence, whether beneficial or detrimental, on Overall Equipment Efficiency (OEE) resulting from operational modifications to the packaging line will have an impact on the operational effectiveness and productivity of production sites.

### Productivity in the Pharmaceutical Business

The University of St. Gallen has conducted multiple studies on the productivity and operations of the pharmaceutical sector from 2005 to the present [30, 32, 43]. Nevertheless, there has been a lack of substantial progress in the productivity of the pharmaceutical business over this time. In the period from 2000 to 2009, Spector [] found that the pharmaceutical sector had a minimal effect on inventory levels when compared to other manufacturing industries. An examination of public corporate data from 2007 to the present reveals that the rate at which inventory is being sold and replaced has been relatively constant, with no significant changes. Basu et al. [45] similarly observed a lack of change in the proportion of total sales spent on items in the pharmaceutical industry from 2006 to 2008. Vernon et al. [46] found that there is a direct correlation between the cost of commodities in medication manufacturing and the cost of healthcare. Manufacturers often retain any decrease in the cost of items as extra profit, while they pass on any increase in the cost of goods to consumers by raising prices [46]. If serialization processes have an impact on productivity, it is possible that this would be seen in the expenses of items and the healthcare expenses for patients.

### Gaps in the Literature

The existing literature on the effects of serialisation on operational efficiency and productivity in the global environment is limited. The literature offered foundational information regarding the perspectives of policymakers and industry leaders regarding the anticipated financial expenses associated with serialisation, including both capital and operating costs. There was limited investigation in the literature about the correctness of the initial expectations indicated in impact assessment studies conducted by policymakers. No literature was located that provided a comprehensive analysis of the specific effects of serialisation on operational efficiency or productivity. Additionally, the literature did not identify the influence of serialisation processes on pharmaceutical production or the cost of goods sold.

## Methodology

Following a review of the literature a research methodology was designed that sought data and input from Irish pharmaceutical manufacturing sites. It would be virtually impossible to get access to a serialisation pack line to determine how serialisation processes impact efficiency and productivity as Pharmaceutical companies by their nature are secretive [] so a qualitative study with interviews was carried out. A qualitative research approach was utilised as it allowed subject to be studied in their own surroundings [47]. Qualitative interviews were also chosen over surveys as they provide a rich source of data that can be expanded upon [48]. In order to develop qualitative interview process was broken into three sections. The first section was designed to understand the participant’s background and to determine the serialisation resources available at the company and to ascertain the site’s actual serialisation experience. The wider impacts of serialisation infrastructure on IT systems, databases, communication interfaces, regulatory departments, design, and supply chain would also not be adequately considered.

The second section of the semi structured interview process examined the impact of serialisation on operational efficiency by discussing OEE measurements and line availability measurements. Participants were asked to share their experiences of serialisation and OEE measurements and comment on their pack line availability.

The final part of the assessment dealt with the serialisation and productivity. Participants were asked about the impact of serialisation on the Cost of Goods Sold (COGS) which ultimately affects the prices paid by the customer. The final section of the interviews and interviewees also examined the relationship between average batch size, serialisation, and productivity. All fifteen questions were developed as a result of the literature review (Table 1).

**Table 1:** Questions

Questions (Non demographic)	Literature sources
1. Do you measure OEE in your site? 2. If so, how is OEE measured? 3. How did you manage serialisation in your pack lines, retrofit or buy new? 4. How many pack lines have you in your site? 5. What were your estimated OEE losses over a 3-month, 3–12 month and > 1 year after implementing serialisation? 6. Were there any factors that caused OEE to improve post serialisation? 7. How often do you have to update your serialisation software/equipment/lines? 8. Can you provide or calculate a cost per pack increase since serialisation? 9. Do you measure line availability arising from serialisation line upgrades? 10. When did you start your serialisation projects? 11. What labour costs have you had as a result of serialisation? 12. Have you an in house Lean or Operational Excellence program? 13. Have you been reducing batch sizes? 14. If so, have operational excellence programs had pushed your companies to produce more just-in-time orders, to reduce batch sizes and decrease inventory positions? 15. Has serialisation exacerbated inefficiencies associated with small batches?	[8, 13, 26, 30, 38, 42, 45, 46, 50–52]

The qualitative interview model development consisted of 4 phases 1. Preliminary phase, 2. Questionnaire development, 3. Pilot testing and 4. Evaluation [49].

Data from the research was collated and the experience of different manufacturing sites was examined to create a series of findings.

For the purposes of this study the researcher connected with the interviewees in advance of the semi-structured interview process to make them aware of the request for interview and the general topic for discussion. The semi structured interview method was suitable for use with a low respondent population [48]. The Irish Health Products Regulatory Authority (HPRA) list 127 companies in Ireland with pharmaceutical manufacturing licenses [53]. Of these its estimated that less than 17 sites have machine-based packing lines with serialisation activities. This study interviewed respondents from 11 companies, so the sample size represents approximately 65% of the pharmaceutical packing sites in Ireland. The information related to the interviewees and company size is organised in Table 2 below.

**Table 2:** Interviewee information

No	Interviewee Title	Company Size
1	Global Serialisation Leader	Large Enterprise (> 50 million packs per annum)
2	Local Serialisation Subject Matter Expert	Large Enterprise (> 50 million packs per annum)
3	Director of Engineering	Large Enterprise (> 20 million packs per annum)
4	Capex Project Manager	Large Enterprise (50 million packs per annum)
5	OpEx Expert	Large Enterprise (50 million packs per annum)
6	Global Serialisation Leader	Large Enterprise (> 20 million packs per annum)
7	Local Serialisation Subject Matter Expert	Large Enterprise (50 million packs per annum)
8	Global Serialisation Leader	Large Enterprise (> 20 million packs per annum)
9	Director of Engineering	Large Enterprise (> 20 million packs per annum)
10	Director of Engineering	Large Enterprise (> 20 million packs per annum)
11	Finance Manager	Large Enterprise (> 20 million packs per annum)
12	Global Serialization Leader	Large Enterprise (50 million packs per annum)
13	Local Serialisation Leader	Large Enterprise (50 million packs per annum)

The interviews were held online with a list of questions provided in advance so that interviews could gather historical data in relation to costs and line performance. Thirteen interviews were considered enough as after 10–12 interviews no new themes were emerging and the data was considered saturated [54].

At the start of the interview process it was stated clearly to interviewees that their confidentiality was assured and that their anonymity would be preserved [55]. A method of pseudonymity and P numbers is used to protect the identity of the participants and their employers.

The data received through the interviews were manually coded using an Excel Spreadsheet. A coding frame was used to identify and tabulate key words and recurring themes with all authors involved[56].

## Results

### Demographic Backgrounds

The researchers interviewed 13 representatives from 11 pharmaceutical companies. The participating companies provided a strong representative sample of the Irish pharmaceutical packing industry, which makes up approximately 65% of the major pharmaceutical packing sites in Ireland. Most of the sites interviewed were large-volume sites, producing over 20 million pharmaceutical packages per annum or producing over 50 million packs per annum [57]. The respondents to the interviews all had direct experience with serialisation implementation and operations and held senior roles in Operations, Engineering, Operational Excellence and Information Technology. 85% of interviewees had over 10 years’ experience in the Pharmaceutical industry and 60% had greater than 15 years’ experience. Participants held positions ranging from global serialisation leaders, local serialisation subject matter experts (SME), director of engineering, capex project managers and OpEx experts. In total the interviewees organisations represented a total of 114 packing lines with 59% of the pack lines discussed having track and trace capabilities.

### Serialisation and Operational Efficiency—OEE Measures, Pack Line Setup and Serialisation

All except one site involved in the study measured OEE. Of the 13 industry professionals that took part in the interviews all expressed an opinion that the OEE of their pack lines was adversely affected by serialisation. Only one site had a fully automated OEE system across all its pack lines but even at this site there was a necessity to produce manual reports from the automated data capture system. Two other sites had one or two pack machines that were capable of measuring OEE automatically, but for the most part these sites still operated on manual OEE data gathering and calculations.

89% of the sites interviewed conducted manual OEE data gathering and calculations. However, these sites had plans to move to automatic OEE capture with OSI PI (Oil Systems Incorporated Plant Information), OEE Systems and Werum mentioned as potential partners for automated OEE data capture. However, due to many other projects being prioritised the timelines to move were unclear in many cases.

Most pack-lines discussed in the interviews were retrofitted. One participant described “*serialisation as trying to modify a car while it is still in motion*” (P3). One quarter of the companies interviewed has 100% retrofitted their pack lines to prepare for serialisation. In comparison just one company purchased entirely new pack-line equipment for the serialisation project. Most companies needed to “*interweave*” (P2) and “*integrate*” (P3-9, 11–13) the deployment of serialisation processes into existing pack-line operations without disrupting production.

The process of retrofitting pack lines for serialisation was cited by many as a “*loss of line availability*” (P 1–6, 8–12) with lines unavailable for production due to serialisation upgrades and had a negative impact on site productivity. However new pack lines with inbuilt serialisation systems were tested and validated at the OEM’s premises without affecting existing operations.

The interviews revealed that the average age of a pack line among participating companies was 11 years. Pack line equipment generally has a lifecycle of 20–25 years[41]. This means that over the next 10 years companies will need to replace pack machines resulting in farther availability losses (Table 3).

**Table 3:** OEE losses

Month	ISPE Article %	Company No						
		3	4	5	6	9	10	11
1–3	5–10	15	50	15	11	0	20	30
3–12	3–8	12	15	5	5	0	7	20
> 12	1–5	10	2	2	3	0	2	0

Table 2 shows that that nearly half of the participating companies could not describe a loss of OEE post serialisation. Even though most companies (90%) indicated that they record OEE only 50% could indicate if serialisation had any impact on efficiency. It may have been that a serialisation SME may not have OEE data readily available. However, in at least two companies, an operational excellence respondent was not able to provide pre and post serialisation data even though the company manually recorded OEE data. This result may indicate that companies had not considered the impact on OEE sufficiently in advance of the serialisation project.

The average OEE loss reported by the companies interviewed was 2.71%. Two companies reported having no loss in OEE despite reporting additional time spent online changeovers. Removing these two companies from the data yields an average negative OEE impact of 3.8%. In those companies that did indicate post serialisation OEE measurements, one company did cite a significant loss to OEE. Company # 4 in Table 2 is a high-volume manufacturer. The company reported a 15% loss to OEE in the 90-day period post serialisation. The ISPE report had predicted a maximum loss of just 10%. In the period up to 12 months post serialisation the ISPE report predicted a maximum loss of 5% while this manufacture experienced a loss of 12% whereas the ISPE report predicted a maximum loss of just 5% after 12 months. This manufacturer continued to see a loss of 10% compared to its pre-serialisation situation. In the period up to 12 months post serialisation the ISPE report had underestimated the losses for this manufacturer by 50%. One-year post serialisation the ISPE report had underestimated the losses for this manufacturer by 100%. This data was deemed accurate as the manufacturer outlined how the company had taken great care to formalise its OEE measurements in the run up to the serialisation project. The company established a “*level playing field*” (P4) across all its manufacturing sites to properly define and measure the potential impact on OEE and had a global team available to monitor the OEE impact of serialisation. Of all the companies interviewed, the company with the highest reported losses seemed to have the most robust, benchmarked, OEE measurement process.

Company #4 reported that after 12 months its OEE figures were in line with the ISPE report with an OEE loss of just 2%. Company #4 initial losses 90 days post serialisation were 5 × times that indicated by the ISPE figures. In the 3-month-to-12-month period post serialisation losses were double for company 4 that expected from the ISPE study while article Company #5 and #6 were broadly in line with the ISPE report expectations.

Company #1 did not have post serialisation OEE results. However, in the interview the company reported that they experienced “*reworks due to serialisation errors about 15 times per year*” (P1). They further elaborated on the “*complexity of reworks involving serialised products to process… with each rework takes 4* × *staff and 3* × *days labour to process*” (P1). In terms of availability this equates to 45 days lost production on one pack line per annum or a 18.75% loss in availability if the site operated on a single shift per day. Across the site in question the rework issue “*equates to a 2% loss of availability across all pack lines*” (P1). This does not consider the time taken for line setup challenges, additional documentation to complete batches or other errors associated with serialisation. One contributor described the serialisation process as “*adding between 10–15 min to each batch setup*” (P5). The same company described testing of the data matrix code scan quality every 15 min. They elaborated that “*If a single unit fails, then all product going back to the last positive test must be removed for testing. At a line speed of 100 parts per minute, 1500 products may need to be tested due to a print error on a single package*” (P5).

Company # 2, which does not measure OEE as key performance indicator (KPI), described a product recall situation because an additional set of characters included in the 2D matrix code “*did not scan at the distribution centre… and this meant that the entire batch need to be reworked*” (P2). Even if these type of quality errors are infrequent the increased risk posed by the serialisation processes should not be ignored.

Company # 5 described the use of challenge materials to check cameras on the serialisation system. These challenge tests and associated paperwork increase the setup time for each batch.

Company #6 reported that initially post serialisation implementation that “*setup times for serialisation increased to 45 min*” (P6). Over time the serialisation setup time has reduced to 20 min per batch. The company described doing approximately 1000 serialised batches per year. They calculated that “*this equates to 333 h per annum for serialisation batch setup, across 6* × *serialised lines on one shift this equates to a 2.89% OEE loss and equates to a company reported loss of 3% loss in OEE due to serialisation*”(P6). However, in addition company #6 also described a loss of line speed due to serialisation “of between 5–10%”.

Company # 10 reported that OEE was impacted by 2% post serialisation. The interviewee described how “*line speeds needed to be reduced by 10% to 15% to ensure that print quality was acceptable for certain products which was dependent on the quality of packaging materials*”(P10). They extrapolated on how poorer quality packaging would require a reduction in line speed for inkjet printers to produce acceptable 2D matrix codes and that machine vibrations could impact the ability of laser printers to print correctly. The quality of glue used in tamper evident seals could cause divots and bumps on cartons that would cause print issues. They also described how serialisation was less forgiving on printers and vision systems than in the period prior to serialisation. The company also described how In Process Control (IPC) checks “*would identify print errors in the 2D matrix codes but that then poor print quality would need to be traced back to the last good unit printed*”(P10). Thus, in the 30-min period between IPC checks “*there was potential for up to 6,000 units to be removed from the line for visual inspection or rework*”. The company also described how setup time for each batch increased by 50% due to serialisation processes. For simple batch changeovers, changeover time went from 30 to 45 min. When container sizes were changed the change-over time went from 2.5 h to 3 h due to line running challenges following line adjustments.

Company # 11 also reported that serialisation processes increased changeover times by 15 to 20 min to allow for line vision challenge tests and documentation.

Company # 9 reported that serialisation processes did not impact their OEE. However, the company did report that “*their line speeds were optimised for packaging quality and indicated that line speeds were reduced to cope with the demands of effectively printing 2D codes*” (P9). The company also reported additional batch setup time but no impact on OEE.

### Factors Affecting OEE Improvement Post Serialisation

Participants cited several common factors between them for improvements in OEE post serialisation. Operator training, the development of subject matter experts and knowledge sharing between pack-line teams all contributed to better pack-line effectiveness. Better operating procedures and software improvements from vendors were also cited as routes to efficiency gains. One high volume site that experienced a high degree of OEE loss went as far as to develop its own ink formulation for the inkjet printers used in serialisation to improve print quality on the data matrix codes.

### Serialisation and Line Availability

**Figure 1:**
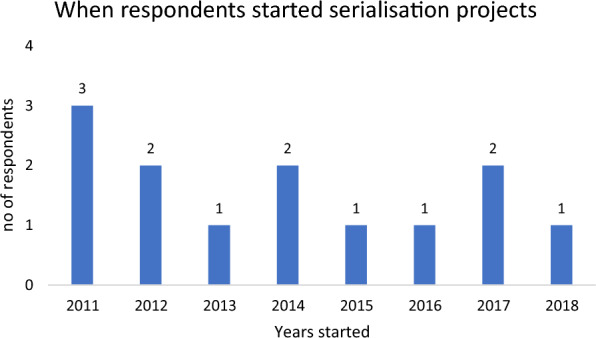
Start of serialisation projects.

Serialisation should not be as a once-off project. Since the introduction of serialisation by the U.S. and European regulatory authorities there has been a series of upgrades and new regulatory milestones. Besides the FDA and EMA regulations, serialisation regulations are also provided by regulators from Turkey, China, Brazil, Saudi Arabia, and Russia. Each new market may have its own requirements and will necessitate serialisation vendors to provide software patches and upgrades. Among the customers interviewed the first serialisation project started in 2011. One company did not start its serialisation project until 2018, just ahead of the European falsified medicines directive deadline in February 2019 (Fig. 1).

Serialisation SME’s described how they are often challenged by team members in operations, line scheduling and OpEx about the impact of serialisation and aggregation online availability as serialisation is deemed to be negative for productivity and line availability by their stakeholders. During the interviews participants were asked how often serialisation systems needed to be updated. 55% of the companies’ interviewed have upgraded their lines for serialisation at least once per year with 85% of participants upgrading lines at least once every eighteen months. In the interviews participants described how line upgrades take between 2 weeks and six weeks to implement. Major upgrades such as the addition of aggregation capabilities can take between three to six months to install. When lines come out of production to undergo these upgrades the loss of availability is not accounted for in OEE calculations rather it is tagged as planned downtime.

**Figure 2:**
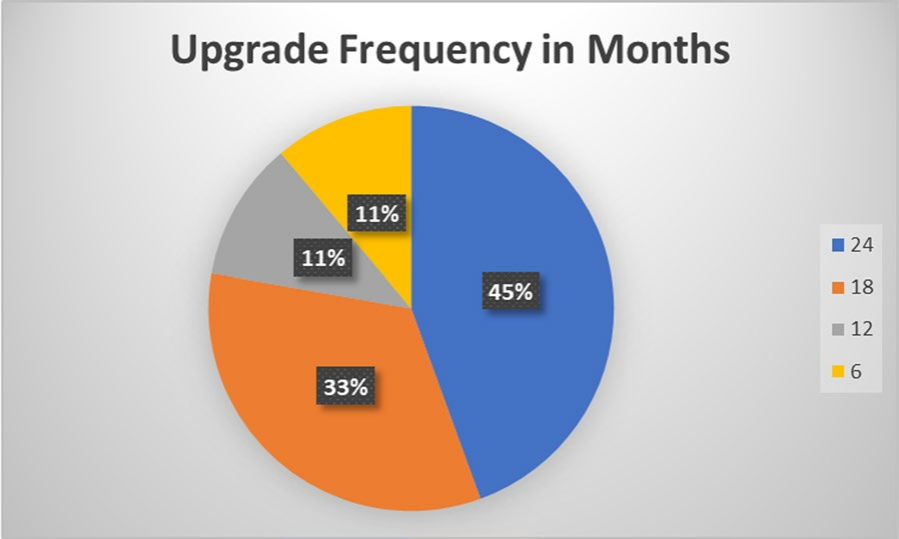
Frequency of line upgrade work (in months).

Bragli et al. have proposed a modification to the OEE calculation to account for the loss of availability due to planned maintenance events. The Overall Equipment Effectiveness Manufacturing (OEEM) measurement uses the standard OEE measurement and multiples by a factor A_pm_, which is the loss of availability due to planned maintenance [58]. Only one of the companies interviewed used OEEM or Equipment Effectiveness as a measure of line availability. However, three sites did describe other measures used to capture the loss of line availability. These measures included calculations for line utilisation, max capacity v’s actual production and site potential capacity calculations. Company #10 had calculated that taking one pack-line off the production schedule for one week equated to a loss of 570,000 units or a 1.14% loss in annual production capacity. The same company reported that this type of line upgrade was required once per year. Figure 2 below records the frequency at which participants make pack lines unavailable so that upgrade work can be carried out.

Companies #1, #6 and #9 reported that line upgrade work resulted in additional evening and weekend shifts, creating additional cost to the business. 55% of the companies interviewed reported that upgrade work takes approximately two weeks. Most of this time is taken up with qualification, validation and change control processes. The addition of aggregation functionality is a major upgrade program. Company #1 reported that an aggregation project would mean a line becoming unavailable for production for between 3 and 6 months.

### Serialisation and Productivity

Four companies in the interviews were able to identify a cost per pack associated with serialisation. Cost to serialise a pack ranged from 2.5 cents to 6 cents. The average cost was 4.1 cents. This cost aligns with the 5 cent per pack outlined in the 2008 European Commission report [19]. The Commission report uses a cost of 2 cent per pack after 5 years presumably because of depreciation.

Based on the volumes of production provided in the interviews and interviews the companies which identified their cost per pack to serialise would have the following annual costs outlined in Table 4.

**Table 4:** Serialisation costs per pack

Company	Cost per pack	Est. annual units	Cost
Company # 3	2.5c	5 m	€125 K
Company # 5	6c	60 m	€3.6 m
Company # 7	5c	90 m	€4.5 m
Company # 11	3c	50 m	€1.5 m

Participants were asked if the additional costs associated with serialisation were reflected in the cost of goods or unit cost per item produced. Four of the companies interviewed were able to provide a percentage impact on unit costs (Table 5).

**Table 5:** Impact on cost of goods/unit cost

Company	% increase in COGS/unit cost
Company #10	1.9
Company #5	0.5
Company #3	2.5
Company #7	5.4

The average increase in cost of goods reported was 2.6%. In the case of company #7 and company #10 the increase in cost of goods is conservative. The figure was calculated using the depreciation cost associated with serialisation equipment only. It does not consider the labour costs associated with running a serialisation line. The use of depreciation cost is particularly useful when examining the impact of serialisation. Four of the companies interviewed still considers their serialisation process to be in the project phase. Even though serialised lines are in operation, ongoing upgrade work means that project resources are still applied. The costs associated with serialisation can be difficult to calculate as budgets are still split between capital projects, operations, local and corporate company structures. Depreciation however cuts across both capital and operational budgets. By using depreciation costs the researcher was able to calculate a serialisation depreciation factor for productivity calculations. The serialisation depreciation factor SD_f_ is calculated as:

$${SD}_{f}=\frac{Annual Depreciation cost}{Unit cost \times units produced per annum}$$

The serialisation depreciation factor provides a simple method to capture part of the costs of serialisation as projects move from initial installation through to business as usual (BAU).

Interviews participants were shown the prediction from the 2008 European Commission report that suggested that the cost of serialisation per pack line at €150,000 per line. Respondents indicated that the cost to serialise a pack line ranged from €250,000 to €900,000 depending on the functionality required. The average cost to serialise a pack line was estimated at €600,000, which four-fold what the E.C. impact report suggested.

These high capital costs have a direct impact on the future operations of the pharmaceutical sites. The capital outlay on serialisation equipment creates a depreciation weight on operational budgets. One participant described serialisation as “*having no benefit to the business and created a cost that was difficult to pass on to contracted customers*”(P13). Another respondent described how “*the weight of depreciation on the budget was restricting the site’s ability to invest in other equipment and that the company was unable to pass on the additional costs to their corporation*”(P9).

Participants were also asked about the ongoing operational costs associated with serialisation. Additional operational costs might include labour costs, data costs, regulatory registration costs and code costs for markets such as China and Russia. While 91% of the companies interviewed cited that serialisation did require additional labour costs, only 55% were able to calculate what the additional labour cost was for their organization. The 2013 report by Pew Charitable Trusts and Booz Hamilton [] predicted an additional labour cost to manufacturers of €242,000 per annum. The participants in this study averaged the additional cost of labour at €88,000 per pack line.

Utilising a conservative calculation of an average increase in the cost of goods of 2.7% the impact of serialisation can be assessed across the Irish pharmaceutical market. The Central Statistics office (CSO) in Ireland reported that in 2019 Ireland exported €15.9bn of packaged pharmaceutical goods. Some of these packaged pharmaceuticals will be filled into their primary packaging containers for export to packing sites outside of Ireland. The CSO does not distinguish between a pharmaceutical as primary packaging goods or as fully finished secondary packaged goods. If we calculate that one third of exports are in their secondary packaging format and that COGs makes up 25% of sale price [66] then a 2.7% increase in the unit cost is valued at €36 m per annum. If intra-company pricing is used for the calculation, then the annual cost is expected to be €143 m per annum.

### Lean Program Deployment and Batch Sizes

All the companies interviewed except for one had an in-house lean manufacturing or operational excellence team. When asked if operational excellence programs had pushed their companies to produce more just-in-time orders, to reduce batch sizes and decrease inventory positions, 55% of respondents said that they now processed more batches annually. However, during interviews with participants it was clear that many companies were trying to move away from the pure just in time (JIT) approach. Nine of the eleven companies (81%) reported that they were actively increasing batch sizes to achieve productivity gains. The philosophy behind smaller batch sizes comes from both the internal demands of operational excellence programs to minimise inventories and from market driven demand. One participant described how “*sales and marketing team members needed to understand that pack line machines were built for high volume production rather than small batch runs*”(P8).

### Effect of Serialisation on Batch Size

When asked if serialisation had exacerbated the inefficiencies associated with small batches 55% of respondents agreed and 45% did not. However, among large volume manufacturers 90% of respondents agreed that serialisation processes had put extra strain on changeover times and efficiencies. Company #10 described how “*a batch that might take 10 min to run but one hour to setup*”(P10). Three companies described how they had decoupled upstream filling from the pack lines to allow the pack lines to produce high volume batches more effectively.

## Discussion

This study aimed to assess the effects of serialisation on the operational efficiency and productivity of pharmaceutical sites in Ireland. The research aimed to evaluate the assumptions made by industry bodies and policy makers prior to the deployment of serialisation. Secondly, to evaluate the influence of serialisation on operational efficiency, site productivity, and costs of goods sold.

The study revealed that serialisation has a detrimental effect on operational efficiency at pharmaceutical facilities in Ireland. Prior to the enactment of the Drug Supply Chain Security Act (DSCSA) and the European Falsified Medicines Directive (FMD), there were discussions in the literature suggesting that serialisation could enhance operational efficiency. An argument was made that the use of new equipment and integrated systems may enhance operational effectiveness. These enhancements in efficiency have not materialized in the Irish setting.

Operational Equipment Effectiveness (OEE) is a crucial metric for assessing efficiency. The study revealed that the post-serialization efficiency of the Irish manufacturing locations surveyed in this research have decreased by an average of 2.71%. After accounting for two companies who claimed no negative impact on OEE despite reporting extra time needed for batch changeovers, the study found a 3.8% decrease in OEE.

The analysis indicates that Irish pharmaceutical manufacturing locations have only partially implemented OEE systems. Although 89% of the surveyed websites collected OEE data, it appeared that there was a deficiency in comparing OEE data before serialization. A limited number of pack machines were only documented on many sites. Several sources indicated that there were delays in machine setup owing to serialisation, but they also noted that there was no decrease in Overall Equipment Effectiveness (OEE).

The determination of Overall Equipment Effectiveness (OEE) relies heavily on the availability of production lines. Due to ongoing regulatory changes and the implementation of serialisation in emerging regions, pack lines are frequently taken out of production to undergo upgrade work. The OEE calculation does not account for this decrease in availability, as the machine loss is documented as scheduled downtime. Approximately 55% of the interview participants reported performing upgrade work at intervals of 18 months or less, with an average period of downtime lasting 2 weeks. The implementation of significant upgrades, such as the inclusion of aggregation features, could result in the unavailability of a line for a period ranging from three to six months. The unavailability of these lines, which are designated for upgrade, results in increased labor expenses for the company. Additional production lines must extend their working hours to compensate for the shortfall. Only one third (36%) of the organizations interviewed utilized metrics to capture this large loss of line availability.

Because of the constant modifications of serialisation equipment there is an overlap between capital projects and operations in Irish pharmaceutical sites. It is difficult to track the expenditures connected between capital projects and operations. The Serialisation depreciation factor SDf gives a simple computation to estimate the influence of serialisation on unit cost. Capital costs accrued in the project phase of serialisation are reflected in operational costs through depreciation line items.

The influence of serialisation on unit cost and cost of products is not insignificant. Using the SDf computation the researcher could determine the impact of serialisation on unit cost as an increase of 2.7%. Based on Irish Central Statistics Office [58], [67] estimates, this reflects an increase in the cost of packaged pharmaceutical goods exported from Ireland of between €36 m—€143 m per annum (depending on whether distribution or intra-company pricing is employed). Some participants in the interviews complained that their businesses were expected to shoulder these increased expenditures. It should be emphasized that a disadvantage of the study is that many organizations interviewed still consider their serialisation process to be in the project phase; therefore, it was too soon in some cases to assess the return on investment (ROI); additionally, in this study, the population was rather small. Even when serialised lines are in operation, continuous upgrade work means that project resources are still applied. The expenses associated with serialisation might be difficult to determine as budgets are still split between capital projects, operations, local and corporate firm structures. Research would show that these higher expenses are ultimately transferred onto patients and payer organizations [68]. Some companies had computed a cost per pack for serialisation with the average cost per pack was 4.1 cents. For large volume sites the annual expense of serialisation is enormous amounting to millions of euro per annum.

The investigation also detected a shift in Irish based pharmaceutical sites away from just-in-time manufacturing. 81% of participating organizations said that they were actively seeking to raise batch sizes and restrict their product range to claw back operational efficiency. While no correlation could be found that serialisation aggravated the impact of smaller batches on pack line productivity it was evident that the companies interviewed were intending to increase batch sizes to attain higher efficiency.

The report also looked back on the assessments of policy makers and industry bodies in anticipation of the track and trace laws. From the literature it was evident that little if any attention was paid to the potential impact of serialisation on efficiency and production. Estimations by policy makers on the cost on the costs associated with serialisation were also inadequate. This research indicated that policy makers underestimated the cost of serialisation projects by a factor of four. A European Commission Assessment report (2008) had predicted an average cost of serialisation per pack of 5 cents [41]. This figure aligned with the experience of the interviews participants which reports a 4.1 cents average cost per pack for serialisation. The average cost per pack is a telling indicator as high-volume sites report an annual cost of serialisation of up to €4.5 m per annum.

The 2018 ISPE report used in the interviews was found to be reliable in terms of the experiences of the sites interviewed. The overall result postulated in the report aligned with the experience of participants in the year after serialisation implementation [13]. However, the impact on efficiency immediately after the implementation of serialisation was much more acute in the Irish context. The ISPE article indicated losses of between 10 to 25% for up to 2 months post serialisation implementation with lines may recover to a position 1–5% lower than the original OEE position after about six months [12]. In a discussion with the author of the article, Alfred Penfold, it was determined that these calculations were based on a combination of the Healthcare Packaging serialisation playbook [23], personal experience and input from industry colleagues. Due to the emerging nature of the technology at that time there was not a large amount of supporting literature for the OEE impact claims in the ISPE article. However the industry sources contributing to the ISPE article would have been close to global serialisation roll-out programs.

## Conclusion

This study identified the impact of serialisation on operational efficiency and productivity in Irish pharmaceutical companies pre and post serialisation as well as quantifying the costs of serialisation on pharmaceutical site productivity, pack cost and cost of goods sold. While there were negative effects of serialisation in terms of costs it should be noted that serialisation is positive overall for the industry and in particular the public in terms of access to safe drug products.

This study has theoretical and managerial implications. Firstly, it demonstrated the lack of accurate cost benefit analysis by regulatory authorities in assessing costing the impact of serialisation on manufacturers and ultimately on the cost of medicines. Future policy decisions should take this case study into account and provide a full cost estimation. Also, from a theoretical point of view this study adds to the state of the art as the first of its kind since serialisation was implemented to investigate the costs of serialisation and its impact on Pharmaceutical productivity. Pharma companies can benchmark the study to assess the effects of serialisation on productivity and costs.

A limitation of this study was the limited number of companies who participated in the study, but they represented a large % of Irish Pharmaceutical manufacturing. It was also difficult to get access to confidential financial information in relation to ROI and payback from Serialisation expenses. A further limitation was this study focused on productivity and efficiency losses via OEE. Further research could be to expand this research to global Pharma organisations to investigate further the annual cost of serialisation as well as expanding the focus of other types of measured losses. A study on the effectiveness of the falsified medicines directive would also be insightful in terms of whether the objectives to protect drug supply chains, particularly in developing countries was met.

## Data Availability

Data is available upon request from the authors.
